# The influence of women’s fear, attitudes and beliefs of childbirth on mode and experience of birth

**DOI:** 10.1186/1471-2393-12-55

**Published:** 2012-06-24

**Authors:** Helen M Haines, Christine Rubertsson, Julie F Pallant, Ingegerd Hildingsson

**Affiliations:** 1 Department of Women’s and Children’s Health, Obstetrics and Gynaecology, Uppsala University, 751 85, Uppsala, Sweden; 2 Rural Health Academic Centre, University of Melbourne, 49 Graham St, Shepparton, Victoria, Australia; 3 Department of Health Science, Mid Sweden University, Sundsvall, Sweden; 4 Department of Women’s and Children’s Health, Division of Reproductive and Perinatal Healthcare, Karolinska Institutet, Stockholm, Sweden; 5 Northeast Health, Green St., 3677, Wangaratta, Victoria, Australia

**Keywords:** Pregnancy, Attitudes, Childbirth fear, Cluster analysis, Scale

## Abstract

**Background:**

Women’s fears and attitudes to childbirth may influence the maternity care they receive and the outcomes of birth. This study aimed to develop profiles of women according to their attitudes regarding birth and their levels of childbirth related fear. The association of these profiles with mode and outcomes of birth was explored.

**Methods:**

Prospective longitudinal cohort design with self report questionnaires containing a set of attitudinal statements regarding birth (Birth Attitudes Profile Scale) and a fear of birth scale (FOBS). Pregnant women responded at 18-20 weeks gestation and two months after birth from a regional area of Sweden (n = 386) and a regional area of Australia (n = 123). Cluster analysis was used to identify a set of profiles. Odds ratios (95% CI) were calculated, comparing cluster membership for country of care, pregnancy characteristics, birth experience and outcomes.

**Results:**

Three clusters were identified – ‘*Self determiners’* (clear attitudes about birth including seeing it as a natural process and no childbirth fear), ‘*Take it as it comes’* (no fear of birth and low levels of agreement with any of the attitude statements) and ‘*Fearful*’ (afraid of birth, with concerns for the personal impact of birth including pain and control, safety concerns and low levels of agreement with attitudes relating to women’s freedom of choice or birth as a natural process). At 18 -20 weeks gestation, when compared to the *‘Self determiners’*, women in the ‘*Fearful*’ cluster were more likely to: prefer a caesarean (OR = 3.3 CI: 1.6-6.8), hold less than positive feelings about being pregnant (OR = 3.6 CI: 1.4-9.0), report less than positive feelings about the approaching birth (OR = 7.2 CI: 4.4-12.0) and less than positive feelings about the first weeks with a newborn (OR = 2.0 CI 1.2-3.6). At two months post partum the ‘*Fearful*’ cluster had a greater likelihood of having had an elective caesarean (OR = 5.4 CI 2.1-14.2); they were more likely to have had an epidural if they laboured (OR = 1.9 CI 1.1-3.2) and to experience their labour pain as more intense than women in the other clusters. The ‘*Fearful*’ cluster were more likely to report a negative experience of birth (OR = 1.7 CI 1.02- 2.9). The ‘*Take it as it comes’* cluster had a higher likelihood of an elective caesarean (OR 3.0 CI 1.1-8.0).

**Conclusions:**

In this study three clusters of women were identified. Belonging to the *‘Fearful’* cluster had a negative effect on women’s emotional health during pregnancy and increased the likelihood of a negative birth experience. Both women in the ‘*Take it as it comes’* and the *‘Fearful’* cluster had higher odds of having an elective caesarean compared to women in the *‘Self determiners’.* Understanding women’s attitudes and level of fear may help midwives and doctors to tailor their interactions with women.

## Background

Understanding and responding to women’s beliefs and attitudes during the childbearing period is an important focus of international maternity health policy. The terms ‘woman centred care’ and ‘informed choice’ reflect that in addition to the physiological aspects of pregnancy and birth, there are psychological, psychosexual, and psychosocial aspects unique to the individual life experiences of pregnant women. These must be considered in order to optimise a woman’s birth outcomes and experience [1]. The psychosocial wellbeing of women is now viewed as equally important as her physical wellbeing [2].

In a ‘woman centred’ approach the clinician moves beyond medico/protocol/risk centric care and seeks to better understand the individual woman through ascertaining her attitudes to pregnancy and birth and her particular life situation [3]. Attitudes have been conceptualized using a three-component model: affective, cognitive and behavioural [4]. The affective component consists of positive or negative feelings toward the attitude object; the cognitive part refers to thoughts or beliefs; and the behavioural element represents the actions or intentions to act upon the object. Social psychologists differentiate a belief from an attitude by suggesting that a belief is the probability dimension of a concept – ‘*is its existence probable or improbable?’*[5] An attitude on the other hand, is the ‘evaluative’ dimension of a concept. ‘*Is it good or is it bad?*’ [5]. A change in attitude toward a given concept can result from a change in belief about that concept [5].

The ‘Harsanyi Doctrine’ [6] asserts that differences in individuals' beliefs can be attributed entirely to differences in information [7]. Applying this doctrine to maternity care, it is interesting to consider where, what, how and by whom, information is shared between women and their care givers and what impact this may have on their beliefs and attitudes. A recent study of 1,318 low-risk Canadian women conducted by the University of British Columbia and the Child & Family Research Institute [8,9] illustrates this point. Focusing on attitudes to birth technology, the Canadian study reported that women attending obstetricians were more favourable to the use of birth technology and were less appreciative of women's roles in their own birth. In contrast, women attending midwives reported less favourable views toward the use of technology and were more supportive of the importance of women's roles. Family practice patients' opinions fell between the other two groups. These women could be a self selecting population who choose a particular care giver according to their pre-existing attitudes, or alternatively the attitudes of the women could be influenced by the information they receive from their caregiver.

The determinants of a woman’s attitudes and beliefs are inherently linked to cultural and health system specific influences [10]. In risk-averse biomedical systems of care the woman’s attitudes and beliefs about birth may determine the level of intervention that she actively chooses or passively receives. With the aim of examining changes over time (1987-2000) in women’s expectations and experiences of intrapartum care, the Greater Expectations Study [11] surveyed approximately 1400 pregnant women across several health services in the United Kingdom (UK). It demonstrated that women’s attitudes and expectations had shifted over the thirteen year period from when the original study [12] had been undertaken. The findings showed a relationship between childbirth outcomes and women’s antenatal attitudes. The issue of greatest concern to the authors was the increase in women’s antenatal anxiety about pain and their reduced faith in their ability to cope with labour [11]. Over the same time period there was an increased use of obstetric interventions, especially induction, epidurals and caesarean sections. Mean scores on a scale designed to measure a willingness to accept interventions (‘attitude to intervention’) were significantly higher in 2000 than in 1987. Women who went on to have unplanned caesarean sections or assisted deliveries had significantly higher ‘attitude to intervention’ scores antenatally than women who went on to have unassisted vaginal deliveries. The study suggested that an explanation for this was an increased use of epidurals by women who were positive about interventions [13]. In 2001 an audit report was tabled in the UK as an investigation of the patterns of, and the reasons for, caesarean [14]. This report included women’s responses to a range of attitudes and beliefs about childbirth. The findings indicated that women who preferred caesarean as the mode of birth held attitudes reflecting a belief that birth was not a natural process and that they were concerned about control and pain and safety.

In clinical practice ‘woman centred care’ and ‘informed choice’ have manifested in such practices as the distribution of evidenced based information brochures, client-held medical records, birth plans and formal screening for psychosocial pathology- in particular perinatal depression and domestic violence [15-19]. Despite the rhetoric, women’s individual circumstances, attitudes, beliefs and choices are not necessarily at the centre of the decisions made in regard to her care. The term ‘woman centred care’ is not a commonly used term in Swedish maternity policy. Women’s personal autonomy is politically important, but the concept of ‘informed choice’ is limited by the State– for example under the universal state funded health system women have no freedom to choose their model of maternity care nor mode of birth [20]. In Australia, choice is often limited by the region where a woman accesses care [21].

In addition to the diagnosis of perinatal depression, researchers and clinicians are increasingly recognising the importance of pregnancy-specific anxiety, with fear of childbirth being a sub construct of this anxiety [22]. A clinically significant fear of childbirth is estimated to affect 20 to 25% of pregnant women and the prevalence of severe fear that impacts on daily life is thought to be between 6 and 10% [23-31]. Most of the literature regarding childbirth fear has been focused on Scandinavian populations, however childbirth fear crosses cultural boundaries as studies from Australia [28,29], the UK [30], Switzerland [31], United States [32] and Canada [23] attest. In an effort to understand a woman’s attitude or belief about birth it is important therefore to add the impact of fear to gain a fuller picture.

In 1985, Raphael-Leff published profiles of pregnant women [33] where she described mothers in four categories: ‘Facilitator’, ‘Regulator’, ‘Reciprocator’, and ‘Conflicted’ (Table 1). Her model, which is based on her extensive clinical experience, mother-child observations and survey data, postulates that there is a variety of approaches to pregnancy and early motherhood within and between societies. She describes these as ‘orientations’ and, while other studies have linked particular personality traits to phenomena such as a request for caesarean for non medical reasons [34], Raphael-Leff states clearly that her model is not about personality traits. Different pregnancies and differing circumstances mean that a woman’s orientation may change with each gestation [33,35].

**Table 1:** Raphael- Leff Orientations

**Orientation**	**Description**
‘The ‘Facilitator’	Sees conception as the culmination of her feminine experience. She regards women as uniquely privileged in pregnancy: ‘Russian-doll-like, each carrying the baby as she herself was carried. Thus identified with both her mother and the baby with whom she communes in introspective thought, she resolves to minimise the transition with as natural a birth as possible.’ [35] p 8.
The ‘Regulator’	‘Dreading the pain of childbirth, she plans as 'civilised' a delivery as possible, making use of medical innovations to decrease the damage.’ [35] p 9. An elevated incidence of elective caesareans was seen amongst the women she identified with this orientation. She interpreted this as an indication of a preference for predictability and a way of bypassing the potentially humiliating experience of vaginal birth. [35].
The ‘Reciprocator’	Sees birth as both stressful and exciting. These women tend to take on a ‘wait-and-see’ attitude.
‘Conflicted’	These women shift between the extreme ‘Facilitator’ and ‘Regulator’ orientations. It is difficult for these women to manage both the contradicting feelings of ‘Facilitator’ and ‘Regulator’ and the uncertainty of the outcome [35].

A recent prospective study from Belgium [36], attempted to predict a woman’s childbirth experience using antenatal expectations of birth and the Raphael-Leff model of orientations. While the antenatal expectations of the women clearly predicted their postpartum recollection of intrapartum experiences, the study did not support the independent contribution to birth experience of the Raphael- Leff orientations after obstetric complications were taken into account. There was a suggestion however, that maternal orientations made some contribution to the childbirth experience.

To assist clinicians in their efforts to sensitively and effectively place women at the centre of maternity care, more knowledge is required about how women think about birth and the extent to which they are fearful. Further empirical research therefore is needed to better understand attitudinal profiles in pregnant women and the association this has with their pregnancy outcome and experience.

In this study we aimed to identify profiles of pregnant women based on their attitudes to and beliefs about birth and their levels of childbirth related fear. We aimed to compare pregnancy characteristics, outcomes and experiences of birth between these profiles. Our hypothesis was that women with an elevated fear of birth would emerge as a distinct profile that had poorer pregnancy and birth outcomes than other women.

## Method

This prospective cohort study is part of a broader longitudinal investigation of aspects of pregnancy, birth and early parenting. The data collection constitutes a sample of rural and regional women in Sweden and Australia undertaken during the years 2007 – 2009.

### Participants

The Swedish cohort was drawn from a regional area in the province of Vasternorrland and the Australian cohort came from a northeast regional area in the state of Victoria. Both sites have an annual birth rate of around five hundred per year and a largely homogenous population of non immigrant women. The Swedish group was recruited at routine ultra sound screening in pregnancy week 17-19. Almost all women undertake this examination in Sweden [37], making it an ideal time to access potential participants. A letter with information about the study was sent two weeks prior to the examination. Swedish speaking women with a normal ultrasound were approached by a recruiting midwife and asked if they wanted to participate in the study. The questionnaire was either filled out at the ultrasound ward, or completed at home and returned by a paid postal envelope. In the Australian setting, all women who give birth at the local hospital attend a booking with a midwife at the antenatal clinic between 18 -20 weeks gestation. At this visit those women who were English speaking with a normal 18 week ultrasound result (thus reducing the chances of women with serious foetal anomalies being sent questionnaires) were invited to take part in the study by the booking midwife. Those who agreed received written information, signed a consent form, and were given a questionnaire to either complete on the spot, or take home and return in a reply paid postal envelope. Reminder letters were posted on two occasions to non responders in both settings.

Ethics approval was obtained from respective regional ethics committees in northern Sweden and Wangaratta, Australia as well as from the Mid Sweden University, and The University of Melbourne.

### Questionnaires

Data was collected using self report questionnaires as part of a larger study, investigating women’s’ experiences of pregnancy and birth. In the study reported here data is from 18 -20 weeks gestation and two months after birth. This study includes data from women who answered questions at both time points.

The questionnaire at 18-20 weeks measured attitudes and beliefs regarding birth by determining the strength of women’s agreement/disagreement on a six-point rating scale to twelve personal and four general statements which had been used previously in two large studies from the UK [14,38]. The sixteen attitudinal items were subjected to factor analysis – reported in a previous study [39]. Four subscales were identified: ‘Personal impact of birth’, ‘Birth as a natural event’, Freedom of choice’ and ‘Safety concerns’. As the four subscales are short (less than ten items) the internal consistency of the subscales were assessed using mean inter-item correlations as recommended by Briggs and Cheek [40]. These ranged from 0.31- 0.40 indicating very good internal consistency. The items and reliability statistics of each subscale are shown in Table 2.

**Table 2:** Birth Attitudes Profile Scale

**Subscales[**[39]**]**	**Mean Inter-item correlation**	**‘I would like a birth that…’ [**[14]**]**
**Safety Concerns**	0.31	is the safest option for me
		is the safest option for my baby
		is the least stressful option for my baby
**Personal Impact of Birth**	0.34	is as pain free as possible
		is the least stressful option for me
		will reduce the chances of ’stress’ or ’cough’ incontinence
		will allow me to feel fit and well sooner
		will least affect my future sex life
		will allow me to plan the date my baby is born
		will allow me to feel more in control
**Freedom of choice**	0.40	If a woman wants to have a CS she should be able to have one under any circumstances
		If a woman wants to have a vaginal birth she should be able to have one under any circumstances
**Birth as a natural event**	0.39	Giving birth is a natural process that should not be interfered with unless necessary
		…is as natural as possible

Total scores for each subscale were calculated by adding together the scores for the individual items. High scores indicated strong agreement. The subscales generated from the set of attitudinal items will be referred to throughout the remainder of this manuscript as the Birth Attitudes Profile Scale (BAPS).

Childbirth fear was also measured at 18 -20 weeks, using a Fear of Birth Scale (FOBS) [29]. Women were asked to respond to the question ‘*How do you feel right now about the approaching birth?’* by marking two 100 mm VAS-scales anchored by the words: *worried/ calm*, and *strong fear/no fear*. These two scores were averaged to give a total score. The FOBS demonstrated excellent internal consistency, with a mean inter-item correlation of 0.84.

The other questions in the questionnaire were drawn from previous population based studies of women’s experiences of pregnancy and birth conducted in Australia and Sweden [41,42]. Five-point Likert scales were used to determine physical health, emotional health and previous birth experience. Women’s feelings about the approaching birth and the new-born were measured by their response to the questions: *“How do you feel about the approaching birth?”* and: *“How do you feel when thinking about the first weeks with a new-born baby?”* Five response alternatives ranged from ‘*Very positive*’ to ‘*Very negative*’ with a middle option of ‘*both positive and negative*’. Responses to all the Likert scales were dichotomised to reflect ‘positive’ or ‘less than positive’. Birth preferences were ascertained by asking the question “*If you had the possibility to choose, how would you prefer to give birth*”, with the response alternatives ‘*Vaginal birth’* and ‘*Caesarean’.*

Women were asked at two months post partum about their mode and experiences of birth. These questions had been previously used in Australian and Swedish studies [41,42]. They were asked to indicate the length of their labour in hours by answering the question *“How many hours did your labour last?”* Their perception of labour pain was explored by the questions: *“How much pain did you feel during labour?”* and *“How did you experience this pain?”* This was assessed by marking two seven point scales anchored with the phrases ‘*no pain at all* (1)*’* to ‘*worst pain imaginable* (7)’ and ‘*Very Negative’* (7) to ‘*Very Positive*’ (1).

### Analysis

Statistical analysis was conducted using SPSS for Windows Chicago, IL, USA Version 17. Characteristics of the women from both cohorts were compared using chi square tests. A cluster analysis was conducted on responses to the BAPS and the level of fear, as determined by the FOBS [29]. As cluster analysis is very sensitive to outliers [43], the data was screened and three outlying cases were identified and removed. These cases contained fear scores at the extreme end of the scale and ‘*not thought about*’ responses to all attitudinal questions. Consistent with the procedures described by Shannon [43], a Kappa-mean cluster analysis, forcing a three cluster solution, was applied to z-score transformed responses to each of the four BAPS subscales and the FOBS mean score. Given the exploratory nature of cluster analysis other possible solutions (e.g. 2-cluster, 4-cluster solutions) were also inspected. The three cluster solution was found to offer the most interpretable and clinically meaningful solution. Each cluster was named according to the grouping of its items after discussion and agreement from the authors that these names gave an authentic and easily understood meaning. Demographic characteristics of the three clusters were compared using chi square statistics.

The next step was to calculate crude and adjusted odds ratios with 95% confidence intervals (CI) for the different explanatory variables during pregnancy and birth using the Mantel–Haenszel technique as described by Rothman [44]. Differences in the continuous data outcome variables measuring length of labour and experience of pain were compared across clusters. Due to unequal group sizes and non-normal distributions this was calculated using the Kruskal Wallis test [44].

## Results

### Participation and response

**Figure 1:**
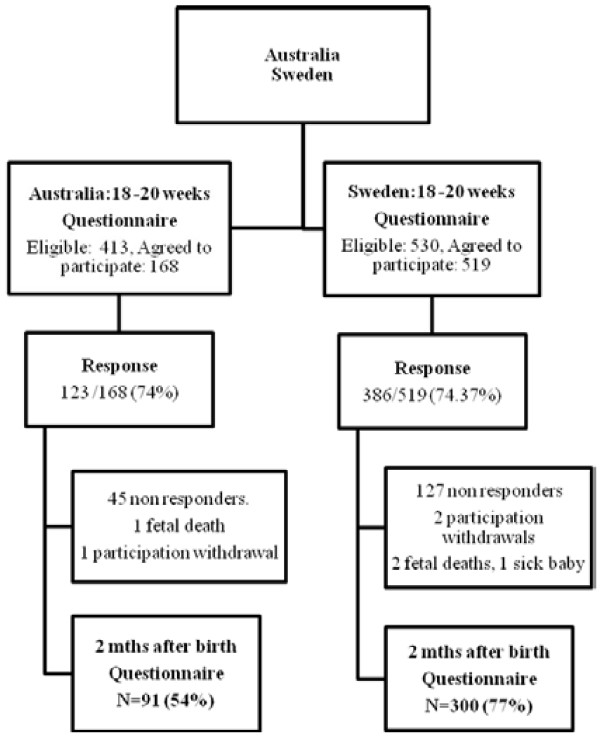
Participation and Response Rates.

Figure 1 shows that of the 530 women who were eligible from the Swedish sample, 519 were recruited, (98% of those eligible), 386 women returned the first questionnaire giving a response rate of 74%. The Australian sample had 413 women eligible, 168 recruited (41% of those eligible) and 123 returns, making a response rate of 74% for the first questionnaire.

At two months post partum a follow-up questionnaire was sent to 386 Swedish women, after exclusion of two intrauterine deaths, one very sick baby, two who withdrew participation and 127 who did not respond to the two first questionnaires. Three hundred post partum questionnaires were completed by the Swedish women. In the Australian cohort the post partum questionnaire was sent to 121 women after the exclusion of 45 women who did not respond to the first questionnaire, one foetal death and one participation withdrawal, leaving 91 women who responded.

### Sample characteristics

The majority of women in both countries were 25 to 35 years old, married or cohabiting and were multiparas (Table 3). The socio demographic characteristics of both samples did not show any statistically significant differences in age, marital status, previous infertility, parity and education. The Australian cohort had significantly more women who had experienced a previous caesarean section; both emergency and elective, while the Swedish cohort had significantly more women who had previously had an instrumental birth (Table 3).

**Table 3:** Characteristics of women in regional cohorts of Australia and Sweden at 18 -20 weeks gestation

	**Australia**	**Sweden**	**X**^**2**^	**DF**	** *p* **
	**n = 123**	**n = 386**			
	**n (%)**	**n (%)**			
**Age**			0.53	2	0.8
<25 years	24 (19.4)	66 (17.1)			
25-35 years	85 (68.5)	270 (69.9)			
>35 years	15 (12.1)	50 (13.0)			
**Married or cohabiting**	116 (95.9)	376 (97.7)	1.2	1	0.30
Not living with a partner	5 (4.2)	9 (2.3)			
**Education**			1.5	1	0.29
Elementary school/high school	61 (51.7)	173 (46.3)			
College/university	57 (48.3)	201 (53.7)			
**Previous Infertility >1 year prior to pregnancy**	17 (13.9)	42 (11)	0.79	1	0.37
**Primiparas**	46 (37.1)	168 (43.5)	1.28	1	0.25
Multiparas	76 (62.3)	218 (56.5)			
**Previous Mode of Childbirth** (Multiparous)					
Vaginal Birth (one or more)	47 (44.6)	172 (36.9)	2.2	1	0.11
Instrumental Vaginal (one or more)	3 (2.5)	39 (10.1)	7.14	1	0.008
Elective caesarean section (one or more)	14 (11.5)	19 (4.9)	6.5	1	0.01
Emergency caesarean section (one or more)	24 (19.7)	26 (6.7)	17.48	1	<0.001

### Cluster analysis

**Figure 2:**
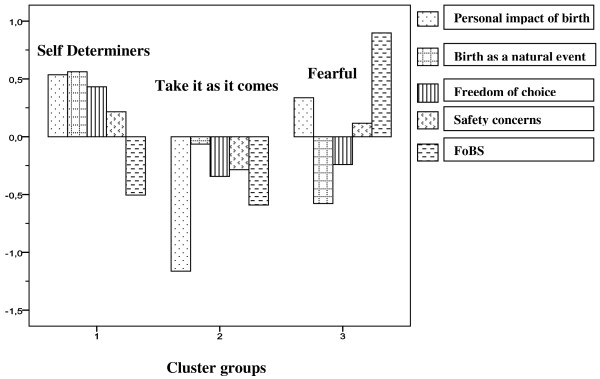
Clusters identified from z-score transformed responses to four attitudinal subscales and FoBs mean score.

Figure 2 shows the three clusters which were identified based on women’s level of agreement to the BAPS items and their level of fear on the FOBS. Cluster 1 ‘*Self determiners’* were characterised by low fear and agreement with the attitudes relating to the personal impact of birth, safety concerns, the natural process of birth and freedom of choice. Cluster 2 ‘Take *it as it comes’ were* not afraid of childbirth. They indicated low levels of agreement on all attitude items. Cluster 3, the *‘Fearful’,* scored high on childbirth fear, showed moderate agreement to the items regarding the personal impact of birth and some concern regarding safety. This group reported low levels of agreement with the items relating to the natural process of birth and to exercising free choice regarding mode of birth.

### Characteristics of clusters

Table 4 shows that the numbers of women in the Australian cohort were evenly spread across the *‘Self determiners’*, *‘Take it as it comes’* and *‘Fearful’* clusters (32%, n = 37, 35%, n = 40, 33%, n = 38 respectively), while the Swedish cohort had a comparatively less balanced membership: *‘Self determiners’* (42%, n = 155), *‘Take it as it comes’* (25%, n = 90), and *‘Fearful’* (33%, n = 121*).* These differences in country of care on cluster membership did not quite reach statistical significance (p < 0.06)*.* The socio-demographic and personal characteristics of each cluster were compared with no differences detected in age, marital status, parity or previous infertility. Women with a lower level of education however, were more likely to belong to the *‘Self determiners’* cluster (p < 0.003), while women who had experienced a previous caesarean were less likely to belong to this group (p < 0.001). Women with a previous negative birth experience were more likely to belong to the *‘Fearful’* cluster (p < 0.001).

**Table 4:** Characteristics of women in the three clusters at 18 -20 weeks

	**Self Determiners**	**Take it as it comes**	**Fearful**	**X**^**2**^**(DF) *p* **
	**n = 192**	**n = 130**	**n = 159**	
	**n (%)**	**n (%)**	**n (%)**	
**Age**				
<25 years	38 (45.8)	25 (29.8)	21 (25.0)	3. 7 (4) 0.44
25-35 years	130 (38.2)	89 (26.2)	121 (35.6)	
>35 years	24 (42.1)	16 (28.1)	17 (29.8)	
**Marital status**				
Living with partner	187 (40.0)	128 (27.4)	153 (32.7)	2.9 (2) 0.23
Not living with partner	4 (36.4)	1 (9.1)	6 (54.5)	
**Education**				
Elementary school/high school	105 (47.5)	52 (23.5)	64 (29.0)	11.5 (2) 0.003
College/university	79 (32.1)	75 (30.1)	92 (37.4)	
Sweden	155 (42.3)	90 (24.6)	121 (33.1)	5.8 (2) 0.06
Australia	37 (32.2)	40 (34.8)	38 (33.0)	
**Infertility >1 year prior to pregnancy**				
Yes	20 (35.7)	16 (28.5)	20 (35.7)	0.46 (2) 0.79
No	171 (40.4)	113 (26.7)	139 (32.48)	
Primiparas	85 (42.1)	49 (24.3)	68 (33.7)	1.4 (2) 0.49
Multiparas	107 (38.4)	81 (29.0)	91 (32.6)	
**Previous caesarean section**				
Yes	11 (10.3)	24 (29.6)	29 (31.9)	15.9 (2) <0.001
No	96 (89.7)	57 (70.4)	62 (68.1)	
**Previous negative birth experience**				
Positive	78 (49.1)	55 (34.6)	26 (16.4)	44.9 (2) <0.001
Less than positive	29 ( 24.2)	26 (21.7)	65 (54.2)	

After adjustment for age, country, education, and parity, Table 5 shows that the ‘*Fearful*’ cluster at 18-20 weeks gestation were more likely to have poorer self rated emotional health than the women in the ‘*Self determiners’* cluster (OR = 3.3 CI 1.5-7.3). They were more likely to prefer a caesarean (OR = 3.3 CI: 1.6-6.8) and more likely to have less than positive feelings about being pregnant (OR = 3.6 CI: 1.4-9.0).This group of women were more likely to report less than positive feelings about the approaching birth (OR = 7.2 CI: 4.4-12.0) and twice as likely to have less than positive feelings about the first weeks with a newborn (OR = 2.0 CI 1.2-3.6).

**Table 5:** Self-rated health, birth preference and feelings about pregnancy, birth and parenting in three clusters of women at 18 -20 weeks

	**Self Determiners**	**Take it as it comes**	**Fearful**		**Fearful**		**Take it as it comes**	
	**(Reference Group)**			**X 2 (DF) *p* **	**Crude Odds Ratios**	**Adjusted Odds Ratios**	**Crude Odds Ratios**	**Adjusted Odds Ratios**
	**n = 192**	**n = 130**	**n = 159**					
	**n (%)**	**n (%)**	**n (%)**		**(95% CI)**	**(95% CI)**	**(95% CI)**	**(95% CI)**
**Self-rated physical health**								
Good/Very good	169 (41.1)	107 (26.2)	131 (32.1)	2.4 (2) 0.29	1.0 ref.	1.0 ref.	1.0 ref.	1.0 ref.
Less than good	23 (31.9)	21 (29.2)	28 (38.9)		1.6 (0.9-2.8)	1.8 (1.0-3.5)	1.4 (0.8-2.7)	1.8 (0.9-3.5)
**Self-rated emotional health**								
Good/Very good	181 (41.8)	117 (27.0)	135 ( 31.2)	7.8 (2) 0.02	1.0 ref.	1.0 ref.	1.0 ref.	1.0 ref.
Less than good	11 (23.9)	12 (26.1)	23 (50.0)		2.8 (1.3-6.0)**	3.3 (1.5-7.3)*	1.7 (0.7-4.0)	1.9 (0.7-4.6)
Vaginal birth	172 (41.4)	116 (27.9)	127 (30.6)	10.8 (2) 0.005	1.0 ref.	1.0 ref.	1.0 ref.	1.0 ref.
Caesarean section	16 (29.1)	10 (18.2)	29 (52.7)		3.5 (1.3-4.7)**	3.3 (1.6 -6.8) ***	0.9 (0.4-2.1)	1.1 (0.4 -2.5)
**Feelings about being pregnant**								
Positive	183 (41.2)	120 (27.0)	141 (31.8)	7.9 (2) 0.02	1.0 ref.	1.0 ref.	1.0 ref.	1.0 ref.
Less than positive	7 (21.2)	8 (24.2)	18 (54.5)		3.3 (1.4-8.2)*	3.6 (1.4-9.0)**	1.7 (0.6-5.0)	1.6 (0.5-4.6)
**Feelings about the approaching birth**								
Positive	139 ( 48.6)	101 (35.3)	46 (16.1)	92.8 (2) 0.001	1.0 ref.	1.0 ref.	1.0 ref.	1.0 ref.
Less than positive	53 (27.5)	28 (14.5)	112 (58.0)		6.4 (4.0-10.0)***	7.2 (4.4-12.0)***	0.7 (0.4-1.2)	0.8 (0.5-1.4)
**Feelings about the first week with a newborn**								
Positive	165 (43.4)	98 (25.8)	117 (30.1 )	9.1 (2) 0.010	1.0 ref.	1.0 ref.	1.0 ref.	1.0 ref.
Less than positive	27 (27.0)	31 (31.0)	42 (42.0)		2.2 (1.3-3.8)**	2.0 (1.2-3.6)*	2.0 (1.1-3.4)*	1.6 (0.9-3.0)

*p < 0.05, **p < 0.01,***p < 0.001.

Adjusted for age, country, education, and parity.

Table 5 shows that at mid pregnancy, the ‘*Take it as it comes*’ cluster had a higher likelihood of having less than positive feelings about the first weeks with a newborn when compared with the ‘*Self determiners’* (OR = 2.0 CI, 1.1-3.4) however this was no longer significant when adjusted for age, country, education, and parity.

### Birth outcomes

At two months post partum Table 6 shows that the women classified in the *‘Self determiners’* cluster had the highest percentage of unassisted vaginal births: 44% (n = 113) compared with 27% (n = 67) in the *‘Fearful’* and 29% (n = 73) in the ‘*Take it as it comes’* cluster (p <0.04)*.* The ‘*Fearful*’ cluster had a greater likelihood of having an elective caesarean (OR = 5.4 CI 2.1 - 14.2) and higher odds of having an epidural if they laboured (OR = 1.9 CI 1.1-3.2). *‘Fearful’* women reported a higher likelihood of having received counselling during pregnancy for their fear of birth when compared with the women in the ‘*Self determiners’* cluster (OR = 5.0 CI 1.9-13.2). Their likelihood of a negative birth experience was higher than for the women in the ‘*Self determiners’* (OR = 1.7 CI 1.01-2.9). At two months post partum (Table 6), the ‘*Take it as it comes’* reported three times the likelihood of elective caesarean OR = 3.0 (CI 1.1-8.0) when compared to the *‘Self determiners’.*

**Table 6:** Birth outcomes in the three clusters 2 months post birth

	**Self Determiners (Reference group)**	**Takes it as it comes**	**Fearful**		**Fearful**		**Take it as it comes**	
	**n = 145**	**n = 102**	**n = 110**	**X2 (DF) p**	**Crude Odds Ratios**	**Adjusted Odds Ratios**	**Crude Odds Ratios**	**Adjusted Odds Ratios**
	**n (%)**	**n (%)**	**n (%)**		**(95% CI)**	**(95% CI)**	**(95% CI)**	**(95% CI)**
**Counselling due to childbirth fear**								
Yes	6 (21.4)	3 ( 10.7)	19 ( 67.8)	19.7 (2) <0.001	4.8 (1.9- 12.6) ***	5.0 (1.9-13.2) ***	0.7 ( 0.7- 2.9)	0.7 (0.2-3.9)
No	139 (42.2)	99 ( 30.1)	91 (27.7)		1.0 Ref.	1.0 Ref.	1.0 Ref.	1.0 Ref.
**Onset of labour (vaginal births only)**								
Spontaneous	119 (45.2)	75 (28.5)	69 ( 26.2)	0.68 (2) 0.71	1.0 Ref.	1.0 Ref.	1.0 Ref.	1.0 Ref.
Induction	24 (42.1)	15 (26.3)	18 (31.5)		1.3 (0.6-2.5)	1.3 (0.6-2.8)	1.0 (0.5-2.0)	0.9 (0.4-2.0)
**Mode of birth**								
Vaginal birth	113 (44.7)	73 (28.8)	67 (26.5)	6.7 (2) 0.04	1.0 Ref.	1.0 Ref.	1.0 Ref.	1.0 Ref.
Instrumental vaginal	15 (48.4)	5 ( 16.1)	11 (35.5)	2.9 (2) 0.24	1.2 (0.5-2.8)	1.0 (0.4- 2.5)	0.5 (0.2-1.5)	0.5 (0.1- 1.4)
Elective caesarean section	7 (17.1)	14 (34.1)	20 (48.8)	12.1 (2) 0.002	4.8 (2.0-12.0)***	5.4 (2.1 - 14.2)***	3.1 (1.2 -8.0) *	3.0 (1.1 - 8.0)*
Emergency caesarean section	14 (33.3)	16 (38.1)	12 (28.5)	1.9 (2) 0.39	1.4 (0.6- 3.3)	1.2 (0.5-2.8)	1.8 (0.8- 3.8)	1.5 (0.6-3.3)
**Epidural**								
Yes	28 (30.8)	25 (27.5)	38 (41.8)	4.9 (2) 0.09	1.8 (1.07-3.2)*	1.9 (1.1-3.2)*	1.4 (0.7-2.5)	1.4 (0.7-2.6)
No	123 (43.9)	83 (29.6)	74 (26.5)	1.0 Ref.	1.0 Ref.	1.0 Ref.	1.0 Ref.	1.0 Ref.
**Birth experience**								
Positive	101 (43.0)	73 (31.1)	61 (25.9)	5.48 (2) 0.06	1.0 Ref.	1.0 Ref.	1.0 Ref.	1.0 Ref.
Less than positive	49 (36.8)	34 (25.6)	50 (37.5)		1.7 (1.02- 2.8 )*	1.7 (1.01-2.9)*	0.9 (0.6- 2.1 )	1.1 (0.6-1.7)
**Baby transferred to NICU**								
Yes	15 (41.7)	11 (30.5)	10 (27.7)	0.09 (2) 0.96	1.1 (0.5 -2.6)	1.0 (0.9 -1.1)	1.0 (0.4 -2.2)	1.1 (0.5 -2.6)
No	135 (40.7)	97 (29.2)	100 (30.1)		1.0 Ref.	1.0 Ref.	1.0 Ref.	1.0 Ref.

*p < 0.05, **p < 0.01,***p < 0.001.

Adjusted for age, education, country and parity.

After excluding women who had an elective caesarean, mean scores were calculated on length, intensity and experience of labour pain across the clusters. The *‘Take it as it comes’* cluster reported a shorter length of labour (p < 0.005) than women in the other two clusters (Table 7). The *‘Fearful’* reported their labour pain as more intense than women in the other clusters (p <0.009). There was no difference between the clusters in the women’s experience of labour pain.

**Table 7:** **Length of labour, pain intensity and pain experieince in women in the three clusters**^** *#* **^

	**Self Determiners**	**Take it as it comes**	**Fearful**	** *p* **
	**n = 145**	**n = 102**	**n = 110**	
	**Mean (SD)**	**Mean (SD)**	**Mean (SD)**	
**Length of Labour (hours)**^**ab**^	10.32 (11.55)	9.07 (8.87)	10.60 (11.52)	0.005
**Pain Intenstity**^**c**^	5.22 (1.28)	5.33 (1.22)	5.65 (1.39)	0.009
**Pain Experienced**^**d**^	3.71 (1.41)	3.93 (1.57)	4.11 (1.73)	0.203

^#^Kruskall-Wallis test.

^a^Elective caesarean sections excluded.

^b^Hours.

^c^7-point rating scale (1 = No pain at all- 7 = Worst pain imaginable).

^d^7-point rating scale (1 = Very positive-7 = Very negative).

## Discussion

This cohort of Swedish and Australian women were categorised into three attitudinal profiles: ‘*Self determiners’, ‘Take it as it comes’* and *‘Fearful’.* Comparison of the women within these clusters revealed differences in emotional health, birth preferences, and feelings about being pregnant. They also showed significant differences in a number of birth outcomes. Of these three profiles, the presence of fear had the most negative impact on women’s emotional health, feelings about pregnancy and parenting and experience of birth. Belonging to the *‘Fearful’* cluster increased a woman’s likelihood of preferring, and actually having, an elective caesarean.

### ‘Fearful’ cluster

The *‘Fearful’* women were characterised by high levels of fear and concerns regarding safety. These women were worried about the personal impacts of birth such as pain, their sense of control and any detrimental effects birth may have on their body. These women did not see birth as a natural event and did not subscribe to an attitude of freedom of choice. Their likelihood of preferring a caesarean was three times that of women in the *‘Self determiners’* cluster. This resonates with the Raphael-Leff‘s description of the *‘Regulator*’ cluster of mothers [33,35,45].

This finding was also consistent with the results of the Thomas and Paranjothy report [14] which described women who preferred a caesarean as more likely to place a high priority on their own safety and being as pain free as possible. Likewise, Thomas and Paranjothy showed that women [14] who preferred caesarean were more likely to disagree with the statement that *‘birth was a natural process that should not be interfered with unless necessary’ -* an attitudinal item included in this *‘Fearful’* cluster group.

It was not surprising to find that the ‘*Fearful’* cluster contained significantly more women with a previous caesarean and a previous negative birth experience. These are well known determinants of childbirth fear [46,47]. Belonging to the *“Fearful’* cluster increased the likelihood of women actualising their preference for an elective caesarean. This higher prevalence of elective caesarean has been described in the literature previously on childbirth related fear from the Nordic populations [42].

Women in the *‘Fearful’* cluster had poorer self rated emotional health in mid pregnancy than women in the other clusters; a finding that points to them being at risk of poor mental health both in the perinatal period and possibly beyond [48]. Women with childbirth related fear are afraid of inadequate support, inability to contribute to important decisions concerning themselves or their baby, losing control and ‘performing’ badly [24-28,31,46,47]. These characteristics again show similarities with Raphael-Leff‘s ‘regulator’ group who see vaginal birth as a potentially humiliating experience [35].

Fear is commonly articulated as fear of unbearable pain, fear for their own and their infant’s safety and fear of obstetric injuries [47]. Women in this cluster reported more negative birth experience than the other clusters. Possibly inherent in their negative experience of birth, was our finding that the *‘Fearful’* cluster of women perceived their labour as more painful than the women in the other clusters. Our findings demonstrated that the *‘Fearful’* cluster had a higher use of epidural. Pain in labour is a complex issue. Despite widespread use of powerful analgesics and modern anaesthetic techniques, many women report high levels of pain with some describing it as the ‘worst pain imaginable’ [49]. Alleviating pain does not guarantee an improvement in women’s experience of labour or their longer term recollections of pain [50].

### ‘Self determiners’ cluster

Overall the *‘Self determiners’* cluster contained the highest proportion of women. These women showed firm opinions on a range of attitudes and beliefs. They were not afraid of childbirth. These women had the highest percentage of unassisted vaginal birth.

The ‘*Self determiners’* were less educated than women in the other two clusters. This finding is in contrast to the media image of the savvy, assertive highly educated woman holding clear views about the type of birth she wants [51]. Likewise it contrasts with the generalisations created by some healthcare professionals who perceive lower educated women as being less informed and less interested in making choices regarding their care. Green et al [52] reported that, contrary to the stereotypes of pregnant women generated by caregivers, the less educated women did not want to hand over all control to the staff and had the highest expectations for a fulfilling birth experience Our findings are commensurate with this.

### ‘Take it as it comes’ cluster

The women in the ‘*Take it as it comes*’ cluster were not afraid of childbirth but they appeared to have no firm attitudinal preferences concerning birth. The ‘*Take it as it comes*’ were no more likely to have preference for either vaginal or caesarean birth than the *‘Self determiners’,* however when actual mode of birth was compared, the ‘*Take it as it comes*’ group had an increased likelihood of elective caesarean. We might postulate that these women will just *‘go with the flow’* as described by Pilley Edwards [53]. The reluctance of some women to engage in autonomous obstetric decision-making has been described and explained in regard to actively choosing mode of birth [38]. Many women feel unable or unwilling to exercise choice regarding mode of birth as any decision is always governed by what is best for the baby in the particular circumstances they find themselves in [38].

This approach is in keeping with Lehman’s (1950) ‘Decision Theory’ as cited by Lie [54] where “there is a certain relationship between a rational person's preferences for acts, probability assignment for states and utility assignment for consequences [54]”. It follows that given most women agree strongly with the paramount importance of safety of the baby, that this *‘Take it as it comes’* group would be particularly vulnerable to acceding to an intervention that was in any way couched with language promoting infant wellbeing. This cluster of women show some characteristics in common with Raphael-Leff’s ‘reciprocator’ orientation who do not have a precise birth ‘plan’, instead holding a ‘wait-and-see’ attitude regarding the childbirth [33].

### Clinical implications

Knowing that information shapes beliefs and can lead to attitude changes [5,6], midwives and doctors have an important role in influencing positive, healthy attitudes to birth in women by providing clear, evidence based information. In caring for women who fit the *‘Fearful’* cluster the findings of this study can assist clinicians to focus on raising discussion about the personal impact of birth. In particular, discussion and planning should address women’s feelings about control and pain. Debunking myths and providing clear communication about risk and safety ought to be a feature of antenatal care. Clinicians have an opportunity to reinforce the natural aspects of the pregnancy and birth experience. Understanding the complexities of the underlying attitudes and fears women bring with them to the antenatal encounter or birthing room can enable maternity-care professionals to interact in a sensitive and meaningful way with women.

Midwives and doctors are in a unique position to develop a trusting insightful relationship with the women they encounter. In being aware of women’s fears in particular, midwives and doctors then must be sensitive to anxieties which can be approached with reassurance, information and one to one support. While the role of specific counselling for fear of childbirth has not been shown to ‘cure’ fear [55,56] clinicians must remain alert to women with serious distress requiring referral for expert psychological help.

Women in the ‘*Take it as it comes*’ cluster may also warrant further attention from clinicians. This group are most likely the women who antenatally seem to have no issues. This group of women could benefit from clear information regarding the potential impacts of intervention on them and their baby. They could be encouraged to take a more proactive approach to giving birth with confident encouragement from their clinician. With clear explanations and guidance from clinicians these women may be potentially positioned to avoid unnecessary intervention.

### Limitations

This study focused on women from two regional areas in Sweden and Australia and, as such, the findings should be interpreted with some caution in terms of generalisablity to other populations. The potential to detect a difference in cluster membership by country of care may have been limited by the relatively low numbers of participants in the Australian cohort. The participation rate in the Australian setting may have been linked to the context of the booking appointment where the women were invited to participate. At this visit the woman may well have been subject to information overload as she is given health promotion information, referrals for blood tests, clinic appointments and antenatal education class information. The burden of completing a questionnaire on top of this may have been too much for some women.

Additional research is needed on a larger number of women to detect if there are systematic differences between the two countries. Further replication of the results of this study across other populations is also needed to confirm their stability, particularly given the exploratory and subjective nature of cluster analysis.

Selection bias is a common problem in the recruitment of participants to cohort studies, as is loss to follow up with a longitudinal design. This study excluded women who were unable to speak the native language of their respective country of care and therefore limited the study’s capacity to explore a more diverse set of opinions and attitudes. Both regional centres are however characterised by low numbers of foreign born women.

The BAPS adopted for this study has shown four subscales measuring attitudes [39] with good internal consistency. The items which constitute the scale have been used in two previous British studies [14,38]. Although the use of a defined set of attitudes limits our ability to identify other salient beliefs that may be relevant, it does allow the responses to be scored and then clustered and compared across groups in a consistent manner. The prospective design of this study ensured that attitudes were measured during pregnancy, thereby avoiding the potential problem with recall bias.

## Conclusion

In this Australian and Swedish study, three clusters of women were identified based on attitudes held during mid pregnancy. Belonging to the *‘Fearful’* cluster had a negative effect on women’s emotional health during pregnancy and increased her likelihood of an operative birth and a negative birth experience. Women in the *‘Take it as it comes’* cluster were identified as a vulnerable group for an operative birth. The results of this study suggest that attitudes and childbirth related fear are important factors related to birth outcome that should be explored by health professionals during the antenatal period. Midwives and doctors can assist women in their preparation for birth by spending time sensitively enquiring about their feelings and attitudes toward pregnancy. Working towards a positive experience of birth is one of the most crucial goals the health team must set. Most especially midwives and doctors must discuss any fears the women may have. Knowledge about women’s attitudes may help midwives and doctors to tailor their interactions with women in such a way as to inform and reassure them in their capacity to give birth and become a mother. The use of this profiling approach on a larger cohort of women is recommended for further research.

## Competing interests

The authors declare that they have no competing interests.

## Authors’ contributions

HH, IH conceived of the study, developed the study instrument and undertook data collection. HH participated in the study design, performed data edits and statistical analyses, wrote the draft, and reviewed and finalized the manuscript. IH participated in the study design, performed data edits, statistical analyses and edited and reviewed the final manuscript. JP participated in the study design, performed data edits, statistical analyses and edited and reviewed the final manuscript. CR participated in the study design, reviewed the study instruments and edited and reviewed the manuscript. All authors read and approved the final manuscript.

## Pre-publication history

The pre-publication history for this paper can be accessed here:

http://www.biomedcentral.com/1471-2393/12/55/prepub
